# Healthcare Continuity in Crisis: Addressing Gaps in Longitudinal Care Through Inferential Statistics

**DOI:** 10.7759/cureus.88127

**Published:** 2025-07-16

**Authors:** Samy Allam, Helena Chan

**Affiliations:** 1 Quality and Health Data Integrity, Arrowhead Regional Medical Center, Colton, USA; 2 Medical Education, California University of Science and Medicine, Colton, USA

**Keywords:** administrative data, clinical relevance, continuity of patient care, healthcare continuity, longitudinal care, outpatient quality metrics, patient quality indicators, patient safety and quality improvement, preventative medicine, transitional care management

## Abstract

Continuity of care is vital to improving outcomes for an aging US population increasingly burdened by chronic conditions. However, systemic fragmentation remains pervasive, exacerbated by disjointed care transitions, misaligned incentives, and inadequate communication across providers and care settings. This study investigates the role of transitional care management (TCM) services, Medicare-reimbursed follow-up visits after hospital discharge, as a potential proxy for continuity of care. Using 2022 publicly available Centers for Medicare and Medicaid Services (CMS) data, the study evaluates whether higher state-level utilization of TCM services correlates with lower rates of potentially avoidable hospitalizations, measured through prevention quality indicators (PQIs). Contrary to the hypothesis, regression analyses revealed a statistically significant positive association between the TCM-discharge ratio and PQI rates in five of 12 indicators, including urinary tract infection, uncontrolled diabetes, and composite PQI measures. This suggests that TCM services may be employed more reactively in high-burden states rather than preventively. Furthermore, urbanization and physician density showed mixed associations with PQIs, while poverty level consistently correlated with higher avoidable hospitalizations across all models, highlighting structural inequities in access and quality. The study’s findings challenge assumptions that increased follow-up care alone reduces hospitalizations. Instead, they suggest that isolated interventions such as TCM are insufficient unless embedded within broader, longitudinal care frameworks. Barriers to continuity identified include the undervaluation of evaluation and management services, lack of cross-provider communication, underuse of claims data for identifying high-risk patients, and fragmentation caused by non-traditional care settings such as urgent care and retail clinics. Meanwhile, opportunities for strengthening care continuity include integrating social determinants of health (SDOH) into clinical care, leveraging health information technology, and enhancing patient trust and engagement. The implications are multifaceted. First, structural drivers such as poverty and health workforce shortages play a more significant role than follow-up alone. Second, policy and reimbursement frameworks must shift toward models that incentivize proactive, coordinated, and relationship-based care. Finally, longitudinal research using patient-level data is needed to better understand causal pathways and inform evidence-based strategies for embedding continuity within evolving healthcare delivery models. This study advances the dialogue on continuity of care by empirically analyzing a national, policy-relevant dataset and drawing attention to the complex interplay among clinical interventions, socioeconomic context, and health system structure. It underscores that continuity is not merely a billing code or a visit count but a system-wide commitment requiring coordinated action from clinicians, payers, and policymakers.

## Introduction

Continuity of care for aging Americans has emerged as a critical concern in an increasingly fragmented healthcare system. With the US population steadily aging, particularly among baby boomers now entering Medicare eligibility, a growing need for consistent and coordinated care extends beyond episodic encounters. These individuals are living longer lives, often while managing complex chronic conditions that demand multidisciplinary oversight, community-based resources, and proactive follow-up. However, gaps in longitudinal care remain pervasive, contributing to preventable hospital readmissions, increased healthcare costs, and suboptimal patient outcomes. This reality underscores the urgency of identifying data-driven interventions that promote sustained, effective care transitions.

This study evaluates the relationship between transitional care management (TCM) follow-up services and broader healthcare quality indicators across US states using publicly available Centers for Medicare and Medicaid Services (CMS) data from 2022. We hypothesized that states with higher utilization of TCM services would have lower rates of avoidable hospitalizations. We analyzed national Medicare physician billing data, prevention quality indicators (PQIs), and state-level healthcare variation metrics to assess whether higher TCM utilization following hospital discharges is associated with improved population health outcomes. Although our correlation analyses revealed unexpected positive associations between TCM utilization and preventable hospitalizations, this foundational study highlights geographic variation that may point to reactive implementation of TCM services. These findings support further investigation into how interventions such as TCM can better align with broader care continuity efforts to reduce preventable hospitalizations.

Barriers to continuity of care

Lack of Identification of Fragmented Care as an Opportunity for Improvement

Reducing fragmentation of care is not an immediate priority in the United States healthcare system because it is a complex issue that requires a consistent, long-term solution involving collaboration among several key healthcare stakeholders, each operating with different incentives and values. Even if one organization adopts new technologies or practices to promote continuity of care or interoperability, its efforts may be limited if surrounding organizations fail to implement similar strategies [1,2]. The discussions below will illustrate how multifaceted the barriers to continuity of care are.

Although continuity of care has been shown to reduce hospitalizations and increase the use of preventive services, systemic challenges continue to hinder its widespread implementation [3-5]. Health systems often evaluate the effectiveness of continuity of care through the lens of financial return on investment via cost savings associated with reduced acute care use. However, in settings where revenue is driven by volume, particularly under fee-for-service payment models, there may be a financial disincentive to reduce utilization. Additionally, some clinicians view continuity of care as a professional value that should be supported systemically and not only rewarded financially [6]. This reveals a deeper misalignment between the value that continuity of care provides to patients and the financial structures under which many health systems operate.

Misconception of Physician-Physician Communication

With the ease of information transfer in everyday technology, many patients and caregivers assume that their health information is seamlessly and automatically shared between providers. While this may occur among providers within the same health system, direct information sharing and explicit discussions between physicians about specific patients happen less frequently than one might expect. Such communication can be time-consuming, and patient data are not always easily accessible [1]. Patients may also be unaware of the regulations under the Health Insurance Portability and Accountability Act (HIPAA), as well as the additional administrative steps that care providers must take to request and access medical records from other institutions.

Communication between physicians across different care settings (e.g., hospitals and outpatient clinics) has historically been challenging, contributing to fragmentation in care transitions and gaps between acute and ongoing community-based care management. Some common failures in physician communication include a lack of standardization in communication practices and low rates of direct physician contact during care transitions [7-9]. The lack of physician-to-physician communication is detrimental to care coordination, as patients may receive conflicting treatment plans from different providers.

Lack of Use of Claims to Identify Patients With Highly Fragmented Care

Claims data refers to health insurance billing information that provides a record of transactions between patients and hospitals, clinics, pharmacies, nursing homes, private practices, and other care facilities. It includes data on services rendered, procedures performed, medications prescribed, charges for services, and payments. Claims data can be a valuable tool for both researchers and providers because it comprises large, relatively accessible datasets with anonymized information that do not require patient authorization for use. However, challenges such as inconsistent coding practices and missing information may discourage researchers and providers from relying on claims data [10,11].

Claims data can be used to analyze continuity of care because a patient’s healthcare and pharmaceutical usage with various providers is generally captured. Additionally, diagnosis-related group (DRG) codes classify patients by health condition, providing predictive capacity regarding utilization based on health status. Fragmentation of care can be assessed by examining patterns such as the time elapsed between provider visits, whether necessary follow-up care is received, and how well services rendered and medications dispensed are coordinated across different providers over an extended period.

Undervaluing of Evaluation and Management Services, Putting Pressure on Primary Care Physicians to See More Patients per Day

Fee-for-service remains the dominant payment model for medical services in the United States. Under this model, reimbursement for procedural appointments tends to be higher than for evaluation and management (E/M) appointments, which focus on diagnosis, treatment, and long-term care. As a result, primary care providers (PCPs) are typically paid less per visit than their specialist or surgical colleagues [12]. To sustain adequate reimbursement, PCPs are incentivized to prioritize patient volume over care quality, leading to shorter appointments, poorer health outcomes, and increased physician burnout [6,13].

PCPs are critical for longitudinal care because they serve as the main coordinators of a patient’s treatment plan. Patients with multiple chronic conditions require support from a PCP to ensure that the treatment plans from their specialty care providers are aligned with their overall care goals, health journey, and capacity. The undervaluing of E/M services, combined with a workforce shortage, poses a major challenge to delivering high-quality continuity of care.

Lack of Consistent Communication Among Community-Based Urgent Care Centers and Patients' Other Physicians

Several recent healthcare industry transformations have contributed to the fragmentation of care. The development of urgent care centers has encouraged some patients to skip regular primary care visits and seek treatment only when acute issues arise [14]. Additionally, retail health clinics and digital health disruptors are increasingly meeting consumer demand for convenience, access, and lower healthcare costs [15,16]. Patients are increasingly choosing alternative avenues for low-acuity primary care services instead of traditional settings, such as medical clinics or hospitals [17-19].

While non-traditional care settings can help increase accessibility to care, they pose a potential barrier to continuity of care. Patients may be less likely to visit their PCPs for routine needs such as vaccinations or annual physical examinations, although many patients view urgent care and retail clinics as a backup rather than a replacement [14,15]. The documentation of care becomes fragmented across different electronic health record systems with limited interoperability. Additionally, non-traditional care settings typically do not follow up with PCPs regarding the encounter, contributing to uncertainty about where a patient is in their care journey. These settings can be effective for addressing isolated acute issues but may disrupt continuity for patients with chronic conditions or acute symptoms that are part of a chronic illness.

Opportunities in continuity of care

Increasing Patient Autonomy, Trust, and Responsibility

Continuity of care represents a comprehensive, longitudinal approach to patient management that spans time and care settings. This model integrates both treatment and preventive services, encouraging coordination among specialties in both inpatient and outpatient environments. Central to this model is the development of strong, sustained relationships between patients and their care teams. As patients engage more consistently with the same providers, they are better positioned to participate in shared decision-making, which leads to increased autonomy and a greater sense of ownership over their health [20,21]. The ongoing nature of these relationships also fosters trust, which is critical for collaboratively managing complex or chronic health challenges. To build trust and improve patients’ confidence in their care teams, clinicians can use shared decision-making to better address patients’ needs and preferences [20,22]. Ultimately, continuity of care establishes a foundation for shared responsibility between patients and clinicians, enhancing adherence to care plans, patient satisfaction, and long-term health outcomes.

Access to Timely Care

In addition to strengthening patient-provider relationships, continuity of care enables timely and effective intervention by ensuring consistent clinical oversight. Regular follow-ups allow for early identification of changes in health status, facilitating prompt responses before conditions escalate. This proactive management is especially critical for chronic diseases, where delays in care can lead to complications and increased use of emergency services [21].

Continuity also supports smoother transitions between care settings and providers, reducing fragmentation [7,9,20]. Importantly, studies have shown that patients’ appreciation for longitudinal care increases over time, particularly when they remain under the care of the same physician [20,21]. Establishing this rapport early, before significant disease progression, ensures that patients can rely on trusted clinicians when care is most urgently needed.

Integrating Social Determinants of Health Into Patient Care

Social determinants of health (SDOH), such as transportation reliability, stable housing, and English-language proficiency, significantly influence a patient’s ability to attend appointments, adhere to treatment plans, and maintain follow-up care. Studies continue to demonstrate that individual determinants have varying degrees of impact on health outcomes, with transportation and language barriers particularly linked to delayed care and fragmented treatment pathways [23-26]. For patients with chronic conditions who frequently use healthcare services, social workers and care coordinators often help navigate these barriers through services such as insurance support, health education, and interpretation.

These efforts naturally align with the goals of continuity of care. Primary care physicians, who already coordinate across specialties, are well-positioned to incorporate SDOH-informed care into ongoing clinical relationships. Research has shown that continuity improves chronic disease management and reduces emergency department visits when paired with social services [3,5,27]. However, although many health systems have adopted screening tools and referral protocols to identify high-risk patients, most interventions remain fragmented and insufficient to meet the full scope of patients’ social needs [23,26,28].

Continuity of care, therefore, offers a valuable platform for integrating social determinants of health into medical care. By leveraging repeated interactions with a consistent care team, longitudinal care models provide a unique opportunity to identify, address, and monitor social needs over time, ultimately improving outcomes and reducing costly acute care episodes [3-5].

Implementation of Available Technology

Continuity of care involves sustained engagement with patients across various care settings and requires clinical coordination among multiple healthcare professionals. Advances in healthcare technology, such as electronic health records (EHRs), patient portals, and secure messaging, facilitate effective communication among providers. Studies have shown that using health technology can improve patient outcomes by reducing hospital readmissions and enhancing patient comprehension of care plans [2,28].

Despite these technological advancements, many health systems remain hesitant to adopt new tools without clear evidence of improved efficiency, financial return, or regulatory alignment. High costs, low patient engagement with technology, and concerns over data privacy continue to limit the use of such systems in clinical settings [1,2,16]. However, the growing complexity of patient populations, such as those with multiple chronic conditions, has created an urgent need for better tools to ensure care continuity. These high-need patients often experience fragmented care and are at greater risk for adverse outcomes, making them a strategic priority for improvement efforts. As a result, their care management needs may catalyze increased technological adoption and innovation, especially in systems transitioning to value-based care models where outcomes and coordination are financially incentivized.

Goals of This Investigation

In this study, we aim to bring longitudinal care to the forefront to address persistent gaps in healthcare delivery and to propose a comprehensive methodology for enhancing care continuity. Our focus is on emphasizing the importance of bridging existing siloed payment models through a deeper understanding of health information exchange. By employing inferential statistics, we demonstrate the critical role that longitudinal care plays in the US healthcare quality framework, which continues to emphasize acute and episodic interventions over preventive measures. This prevailing trend undermines the proactive and holistic approach that is fundamental to effective longitudinal care. Collectively, these barriers hinder the delivery of consistent, patient-centered care, which is essential to improving long-term health outcomes.

## Materials and methods

Research design

This nationwide, population-level observational study evaluates state-level aggregate data across the United States using publicly available CMS datasets from 2022. The study setting encompasses all 50 US states and the District of Columbia, allowing for an assessment of variation in TCM utilization and its association with PQIs, which serve as a proxy for population-level avoidable hospitalization rates [29,30]. This study evaluates the relationship between TCM follow-up services usage [29] and broader healthcare quality indicators across US states using publicly available CMS data for 2022 [30]. We conducted twelve multivariable linear regression analyses to examine the relationship between the ratio of TCM follow-up visits (n = 757,481) to hospital discharges (n = 5,655,838) and avoidable hospitalization rates, using twelve different PQIs at the state level.

To control for potential confounding, we included several demographic covariates, including (a) Medicaid expansion status in 2022 [31]; (b) physician density rate (per 100,000 population) [32]; (c) poverty level (percentage of the population below the federal poverty line) [33]; (d) urbanization rate (percent of the population in urban areas) [34]; (e) median age [35]; and (f) the Health Professional Shortage Area (HPSA) “percent need met” metric [36]. For the urbanization rate, decennial population census data from 2020 were used to estimate the approximate urbanization rate in 2022. All covariate data were sourced from publicly accessible, free-to-use datasets. Collinearity was assessed by verifying that variance inflation factor (VIF) scores were below 4 and that tolerance levels exceeded 0.25 to ensure model stability. Statistical significance was defined as p < 0.05.

Transitional care management to hospital discharge ratio

We used the publicly available, free-to-use Medicare Physician & Other Practitioners - by Provider and Service dataset to identify the number of TCM encounters and hospital discharges in 2022 [29]. The dataset was filtered using the relevant Current Procedural Terminology (CPT) codes. CPT codes used in this analysis are copyrighted by the American Medical Association (AMA). Use of CPT codes in this study complies with the AMA CPT Licensing Agreement, which was accepted prior to accessing the publicly available CMS dataset containing these CPT codes.

TCM services occur during the 30-day period after a physician discharges a patient from an inpatient stay. These services are intended for patients who require moderate or high-complexity medical decision-making during the transition from an inpatient hospital setting to a community-based setting, which includes their home, group home, nursing facility, or assisted living facility [37]. They are coded under CPT codes 99495 and 99496. Studies have shown that the utilization of TCM services reduces patient mortality, healthcare utilization, and the overall cost of care [38-40]. For a visit to be categorized under TCM services, communication with the patient and caregiver must occur within two business days of discharge, and a face-to-face visit must be conducted within 14 or 7 calendar days of discharge for HCPCS codes 99495 and 99496, respectively. Given the requirements for an appointment to qualify as a TCM service, we determined that these codes would serve as relevant indicators for evaluating the rate of follow-up visits.

We compared the number of TCM services (n = 757,481) to the number of hospital discharge day managements (n = 5,655,838) to create a TCM-discharge ratio by state in 2022 [29]. We defined hospital discharges as services rendered under provider CPT codes 99238 and 99239. A higher TCM-discharge ratio suggests that more follow-up visits occur after a patient is discharged, reflecting greater use of continuity of care services. In contrast, a lower TCM-discharge ratio indicates fewer follow-up visits after discharge and less reliance on continuity of care.

One of the limitations of utilizing TCM as a metric is that TCM billing may be underutilized or may vary depending on coding behavior [38]. The TCM CPT codes were developed in 2013 and have payment rates significantly higher than those of regular office visits [41]. However, given their relatively recent introduction compared to traditional office visit CPT codes, physicians and other practitioners may lack awareness of or interest in adjusting their workflows to incorporate these alternative codes, even if the payment rate is higher. Additionally, TCM services represent only one modality of continuity of care during a specific period: hospitalization discharge follow-up. Further analysis to enhance the understanding of continuity of care could include evaluating additional strategies such as participation in chronic care management, consistency of appointments with a specific care team, and comparisons of outcomes between patients lost to follow-up and those who maintain consistent follow-up.

Prevention quality indicators (PQIs)

To evaluate broader healthcare quality indicators, we utilized publicly available, free-to-use PQIs by state, accessed through the Centers for Medicare and Medicaid Services (CMS) Mapping Medicare Disparities by Population interactive tool’s Population View [30]. The Agency for Healthcare Research and Quality (AHRQ) created PQIs by using data from hospital discharges to identify admissions that could have been avoided through access to high-quality outpatient care, measured per 100,000 population [42]. A lower PQI indicates fewer avoidable hospitalizations per 100,000 population, suggesting that patients are receiving timely and effective care in the community. By contrast, a higher PQI indicates a greater rate of hospitalizations per 100,000 population that could have been prevented with better outpatient care. For consistency and comparability across states, we used the age-standardized, smoothed PQI values for 2022. For our analysis, we evaluated the PQIs listed in Table 1.

**Table 1:** Prevention quality indicators (PQIs) in inpatient settings measures created by the Agency for Healthcare Research and Quality (AHRQ) Abbreviations: COPD, chronic obstructive pulmonary disease; PQI, prevention quality indicator. Source: PQI data obtained from the Centers for Medicare and Medicaid Services (CMS) Medicare Mapping Disparities Tool [30]; PQI definitions adapted from the Agency for Healthcare Research and Quality [42].

Prevention Quality Indicator (PQI)	Definition
PQI #1: Diabetes Short-Term Complications	Hospitalizations for a principal diagnosis of diabetes with short-term complications (ketoacidosis, hyperosmolarity, or coma) per 100,000 population, ages 18 years and older. Excludes obstetric hospitalizations and transfers from other institutions.
PQI #3: Diabetes Long-Term Complications	Hospitalizations for a principal diagnosis of diabetes with long-term complications (renal, eye, neurological, circulatory, other specified, or unspecified) per 100,000 population, ages 18 years and older. Excludes obstetric hospitalizations and transfers from other institutions.
PQI #5: COPD or Asthma in Older Adults	Hospitalizations with a principal diagnosis of chronic obstructive pulmonary disease (COPD) or asthma per 100,000 population, ages 40 years and older. Excludes hospitalizations with cystic fibrosis and anomalies of the respiratory system, obstetric hospitalizations, and transfers from other institutions.
PQI #7: Hypertension	Hospitalizations with a principal diagnosis of hypertension per 100,000 population, ages 18 years and older. Excludes hospitalizations with stage 1- 4 or unspecified chronic kidney disease combined with a dialysis access procedure, hospitalizations for cardiac procedure, obstetric hospitalizations, and transfers from other institutions.
PQI #8: Heart Failure	Hospitalizations with a principal diagnosis of heart failure per 100,000 population, ages 18 years and older. Excludes hospitalizations with cardiac procedure, obstetric hospitalizations, and transfers from other institutions.
PQI #11: Community-Acquired Pneumonia	Hospitalizations with a principal diagnosis of community-acquired bacterial pneumonia per 100,000 population, ages 18 years or older. Excludes hospitalizations with sickle cell or hemoglobin-S disease, other indications of immunocompromised state, obstetric hospitalizations, and transfers from other institutions.
PQI #12: Urinary Tract Infection	Hospitalizations with a principal diagnosis of urinary tract infection per 100,000 population, ages 18 years and older. Excludes hospitalizations with a kidney or urinary tract disorder, hospitalizations with other indications of immunocompromised state, obstetric hospitalizations, and transfers from other institutions.
PQI #14: Uncontrolled Diabetes	Hospitalizations for a principal diagnosis of uncontrolled diabetes without mention of short-term (ketoacidosis, hyperosmolarity, or coma) or long-term (renal, eye, neurological, circulatory, other specified, or unspecified) complications per 100,000 population, ages 18 years and older. Excludes obstetric hospitalizations and transfers from other institutions.
PQI #16: Lower-Extremity Amputation Among Patients with Diabetes	Hospitalizations for diabetes and a procedure of lower-extremity amputation (except toe amputations) per 100,000 population, ages 18 years and older. Excludes traumatic lower-extremity amputation hospitalizations, obstetric hospitalizations, and transfers from other institutions.
PQI #90: Prevention Quality Overall Composite	Prevention Quality Indicators (PQI) overall composite per 100,000 population, ages 18 years and older. Includes hospitalizations for one of the following conditions: diabetes with short-term complications, diabetes with long-term complications, uncontrolled diabetes without complications, diabetes with lower-extremity amputation, chronic obstructive pulmonary disease, asthma, hypertension, heart failure, bacterial pneumonia, or urinary tract infection.
PQI #91: Prevention Quality Acute Composite	Prevention Quality Indicators (PQI) composite of acute conditions per 100,000 population, ages 18 years and older. Includes hospitalizations with a principal diagnosis of one of the following conditions: bacterial pneumonia or urinary tract infection.
PQI #92: Prevention Quality Chronic Composite	Prevention Quality Indicators (PQI) composite of chronic conditions per 100,000 population, ages 18 years and older. Includes hospitalizations for one of the following conditions: diabetes with short-term complications, diabetes with long-term complications, uncontrolled diabetes without complications, diabetes with lower-extremity amputation, chronic obstructive pulmonary disease, asthma, hypertension, or heart failure.

## Results

We conducted 12 multilinear regression models to examine the relationship between the TCM-discharge ratio and PQI rates across US states while controlling for potential confounding variables, including age distribution, poverty rate, urbanization, physician density, and Medicaid expansion status. The primary independent variable of interest was the TCM-discharge ratio.

TCM associations

Among the 12 PQIs analyzed, five showed a statistically significant (p < 0.05) positive association with the TCM-discharge ratio: PQI #12 (Urinary Tract Infection), PQI #14 (Uncontrolled Diabetes), PQI #90 (Prevention Quality Overall Composite), PQI #91 (Prevention Quality Acute Composite), and PQI #92 (Prevention Quality Chronic Composite). PQI #5 (Chronic Obstructive Pulmonary Disease or Asthma in Older Adults) and PQI #7 (Hypertension) had borderline statistically significant associations, with p values of 0.064 and 0.058, respectively. A full summary of the regression results is provided in Table 2.

**Table 2:** Regression analysis of the TCM-discharge ratio and prevention quality indicator (PQI) rates across states (2022) *Indicates that the TCM-discharge ratios are statistically significant at p < 0.05. Abbreviations: PQI, prevention quality indicator; TCM, transitional care management; COPD, chronic obstructive pulmonary disease; HPSA, health professional shortage area; HRSA, health resources and services administration Source: TCM and discharge data obtained from Centers for Medicare and Medicaid Services (CMS) [29]; PQI data obtained from CMS [30]; Medicaid expansion status obtained from Kaiser Family Foundation [31]; physician density rate obtained from HRSA [32]; poverty status obtained from US Census Bureau [33]; urbanization rate obtained from US Census Bureau [34]; median age obtained from US Census Bureau [35]; health professional shortage area (HPSA) “percent need met” metric obtained from HRSA data [36]. Note: Use of Current Procedural Terminology (CPT) codes within this dataset is subject to the American Medical Association's CPT Licensing Agreement.

Prevention Quality Indicator (PQI)	Overall Multivariable Linear Regression Model					TCM-Discharge Ratio Analysis		
	R	R^2^	Adjusted R^2^	F-statistic	Significance of the F-test	B (unstandardized)	β (standardized)	P value (significance)
PQI #1: Diabetes Short-Term Complications	0.751	0.563	0.492	7.927	<0.001	0.425	0.147	0.192
PQI #3: Diabetes Long-Term Complications	0.676	0.457	0.369	5.176	<0.001	1.668	0.187	0.138
PQI #5: COPD or Asthma in Older Adults	0.537	0.288	0.172	2.484	0.031	4.938	0.269	0.064
PQI #7: Hypertension	0.769	0.591	0.524	8.878	<0.001	1.722	0.209	0.058
PQI #8: Heart Failure	0.616	0.380	0.279	3.763	0.003	8.839	0.203	0.132
PQI #11: Community-Acquired Pneumonia	0.761	0.578	0.510	8.431	<0.001	2.614	0.105	0.343
PQI #12: Urinary Tract Infection	0.755	0.571	0.501	8.167	<0.001	7.235	0.336	0.004*
PQI #14: Uncontrolled Diabetes	0.716	0.513	0.434	6.473	<0.001	1.243	0.290	0.017*
PQI #16: Lower-Extremity Amputation Among Patients with Diabetes	0.719	0.516	0.438	6.561	<0.001	0.605	0.163	0.171
PQI #90: Prevention Quality Overall Composite	0.701	0.492	0.409	5.945	<0.001	31.394	0.278	0.025*
PQI #91: Prevention Quality Acute Composite	0.769	0.591	0.525	8.889	<0.001	10.356	0.236	0.033*
PQI #92: Prevention Quality Chronic Composite	0.689	0.474	0.389	5.546	<0.001	20.417	0.258	0.040*

As demonstrated in Table 2, a one-unit increase in the TCM-discharge ratio was associated with an increase of 7.2 hospitalizations per 100,000 population for urinary tract infections (PQI #12, β = 0.336, p = 0.004), suggesting a reactive pattern of TCM deployment in higher-burden regions. Contrary to our initial hypothesis, the direction of the associations (unstandardized B and standardized β) was positive in all models, indicating that higher TCM use was associated with higher PQI rates, not lower. This may suggest that TCM services are more heavily utilized in regions with higher rates of avoidable hospitalizations, potentially as a reactive measure rather than a preventive intervention.

The adjusted R² values ranged from approximately 0.17 to 0.52, indicating that the included covariates explained between 17% and 52% of the variability in PQI outcomes. The strongest models were seen in PQI #7 (R² = 0.524), PQI #11 (R² = 0.510), and PQI #91 (R² = 0.525), reflecting more robust associations in those areas.

Covariate associations

In our 12 multivariate linear regressions, we utilized structural characteristics to evaluate potential confounding variables by state, including: Medicaid expansion status, physician population, rate of the population below federal poverty levels, urbanization rate, median age, and percentage of HPSA needs met. In this section, we present tables highlighting covariates with more than three statistically significant associations with respective Prevention Quality Indicators (PQIs), including poverty level, urbanization rate, and physician population. Additional tables for covariates with fewer than three statistically significant relationships (Medicaid expansion status, median age, and percentage of HPSA needs met) are included in the appendix for reference.

Poverty level consistently showed a statistically significant positive association with higher rates of preventable hospitalizations across all PQI values analyzed, as detailed in Table 3. The β values were all positive and ranged from 0.376 to 0.677, indicating a moderate to strong association between poverty and avoidable hospitalizations. As poverty levels increased, PQIs also increased. These findings suggest that poverty may be a key structural driver of poor chronic care outcomes and highlight the importance of targeted interventions in high-poverty areas to improve access, care coordination, and long-term disease management.

**Table 3:** Regression coefficients for poverty level across prevention quality indicators (PQIs) Abbreviations: PQI, prevention quality indicator; COPD, chronic obstructive pulmonary disease; CPT, Current Procedural Terminology; TCM, transitional care management. Source: TCM and discharge data obtained from Centers for Medicare and Medicaid Services (CMS) [29]; PQI data obtained from CMS [30]; poverty status obtained from US Census Bureau [33].

Dependent Variable	B (unstandardized)	Standard Error	β (standardized)	t	Significance	Tolerance	Variance Inflation Factor (VIF)
PQI #1: Diabetes Short-Term Complications	3.732	0.602	0.677	6.193	<0.001	0.849	1.178
PQI #3: Diabetes Long-Term Complications	10.310	2.072	0.607	4.975	<0.001	0.849	1.178
PQI #5: COPD or Asthma in Older Adults	13.134	4.883	0.376	2.690	0.010	0.849	1.178
PQI #7: Hypertension	10.379	1.661	0.662	6.250	<0.001	0.849	1.178
PQI #8: Heart Failure	36.487	10.823	0.439	3.371	0.002	0.849	1.178
PQI #11: Community-Acquired Pneumonia	19.744	5.116	0.415	3.859	<0.001	0.849	1.178
PQI #12: Urinary Tract Infection	25.246	4.454	0.615	5.668	<0.001	0.849	1.178
PQI #14: Uncontrolled Diabetes	4.735	0.944	0.580	5.018	<0.001	0.849	1.178
PQI #16: Lower-Extremity Amputation Among Patients with Diabetes	3.731	0.816	0.526	4.570	<0.001	0.849	1.178
PQI #90: Prevention Quality Overall Composite	129.329	25.380	0.601	5.096	<0.001	0.849	1.178
PQI #91: Prevention Quality Acute Composite	45.635	8.850	0.546	5.157	<0.001	0.849	1.178
PQI #92: Prevention Quality Chronic Composite	81.025	18.089	0.537	4.479	<0.001	0.849	1.178

The urbanization rate was statistically significant in four models: PQI #3 (Diabetes Long-Term Complications), PQI #11 (Community-Acquired Pneumonia), PQI #14 (Uncontrolled Diabetes), and PQI #91 (Prevention Quality Acute Composite), as shown in Table 4. A positive or negative β value indicates whether higher urbanization is associated with an increase or decrease in PQI rates, respectively. The β values showed moderate effect sizes, ranging from -0.394 to 0.318.

**Table 4:** Regression coefficients for urbanization rates across prevention quality indicators (PQIs) Abbreviations: PQI, prevention quality indicator; TCM, transitional care management. Source: TCM and discharge data obtained from Centers for Medicare and Medicaid Services (CMS) [29]; PQI data obtained from CMS [30]; urbanization rate obtained from US Census Bureau [34].

Dependent Variable	B (unstandardized)	Standard Error	β (standardized)	t	Significance	Tolerance	Variance Inflation Factor (VIF)
PQI #3: Diabetes Long-Term Complications	0.940	0.427	0.318	2.203	0.033	0.605	1.652
PQI #11: Community-Acquired Pneumonia	-3.264	1.054	-0.394	-3.096	0.003	0.605	1.652
PQI #14: Uncontrolled Diabetes	0.445	0.194	0.313	2.287	0.027	0.605	1.652
PQI #91: Prevention Quality Acute Composite	-3.891	1.823	-0.267	-2.134	0.039	0.605	1.652

Notably, urbanization rates were positively associated with PQIs related to diabetes (long-term complications and uncontrolled diabetes), meaning that as urbanization increased, avoidable hospitalizations for diabetes also increased. In contrast, urbanization was negatively associated with PQIs for community-acquired pneumonia and the PQI #91 (Prevention Quality Acute Composite), indicating that higher urbanization was linked to fewer acute preventable hospitalizations, such as those for pneumonia. These mixed associations suggest a nuanced relationship between urbanization and PQI indicators, potentially influenced by differences in lifestyle, healthcare access, or the effectiveness of long-term preventive care management.

Finally, the physician population (described as the rate of physicians per 100,000 population) was statistically significant in four PQI models: PQI #5 (Chronic Obstructive Pulmonary Disease or Asthma in Older Adults), PQI #7 (Hypertension), PQI #8 (Heart Failure), and PQI #92 (Prevention Quality Chronic Composite), as detailed in Table 5. The β values were all positive and ranged from 0.308 to 0.381, indicating that a greater population of physicians by state was moderately associated with higher rates of avoidable hospitalizations.

**Table 5:** Regression coefficients for physician population across prevention quality indicators (PQIs) Abbreviations: COPD, chronic obstructive pulmonary disease; PQI, prevention quality indicator; TCM, transitional care management. Source: TCM and discharge data obtained from Centers for Medicare and Medicaid Services (CMS) [29]; PQI data obtained from CMS [30]; urbanization rate obtained from US Census Bureau [34].

Dependent Variable	B (unstandardized)	Standard Error	β (standardized)	t	Significance	Tolerance	Variance Inflation Factor (VIF)
PQI #5: COPD or Asthma in Older Adults	0.213	0.105	0.308	2.040	0.048	0.729	1.372
PQI #7: Hypertension	0.098	0.036	0.314	2.749	0.009	0.729	1.372
PQI #8: Heart Failure	0.589	0.232	0.357	2.541	0.015	0.729	1.372
PQI #92: Prevention Quality Chronic Composite	1.139	0.387	0.381	2.942	0.005	0.729	1.372

## Discussion

Our initial hypothesis was that higher use of TCM services would be associated with lower rates of preventable hospitalizations. Contrary to expectations, our analysis found that states with higher TCM-discharge ratios actually had higher rates of PQIs, a set of metrics developed by the AHRQ that reflect hospitalizations that could have been avoided through access to high-quality outpatient care. This positive association was statistically significant for PQI #12 (Urinary Tract Infection), PQI #14 (Uncontrolled Diabetes), and all three composite PQI scores (acute, chronic, and overall), as shown in Table 2.

One possible explanation for this unexpected finding is that TCM services may be more commonly used as a reactive response to higher hospitalization rates rather than as a proactive preventive strategy. States with higher PQI rates may be implementing TCM to address ongoing gaps in outpatient follow-up or care coordination. However, since our analysis is cross-sectional, we cannot determine whether high PQI rates led to increased TCM usage or whether expanded TCM services failed to reduce hospitalization rates. Future studies would require patient-level or longitudinal data to test whether TCM use precedes reductions in avoidable hospitalizations.

The introduction of TCM codes in January 2013 coincided with major provisions of the Affordable Care Act (ACA) that aimed to improve care coordination and reduce healthcare costs. TCM was designed to help patients transition from inpatient to outpatient care by ensuring follow-up within 30 days of discharge. Previous studies have found that TCM services can reduce healthcare costs, hospital utilization, and even mortality among patients who aim to improve care coordination and reduce healthcare costs [38-40]. Our results suggest, however, that at the state level, increased TCM utilization does not necessarily align with better outcomes, possibly because the service is not consistently reaching patients who would benefit from it most or is not being implemented early enough to prevent readmissions. While TCM services are designed to reduce fragmentation immediately following discharge, they represent just one component of a broader continuity of care model. Our findings suggest that isolated interventions, without consistent, upstream, and longitudinal coordinated care, may have limited impact on reducing preventable hospitalizations.

In addition to TCM usage, our covariate analysis revealed important associations between PQIs and broader structural and environmental factors. Poverty was statistically significantly positively associated with all analyzed PQIs, indicating that as the proportion of a state’s population living below the federal poverty line increased, the rate of avoidable hospitalizations also increased. This finding reinforces how strongly health outcomes are influenced by social determinants of health. Limited access to primary care, lower health literacy, and insurance instability in lower-income communities may contribute to disruptions in care continuity, delays in seeking care, and higher hospitalization rates. These results support the need for both federal and state policies that address poverty-related barriers to care through expanded coverage, improved health education, and the provision of community-based preventive services.

Urbanization and physician density were also significantly associated with several PQIs. Urbanization had mixed effects: it was positively associated with diabetes-related PQIs but negatively associated with some acute care PQIs. This suggests that, while urban settings may provide better acute care infrastructure, they may also contribute to chronic disease burdens due to lifestyle changes, dietary patterns, and environmental exposures [43]. The relationship between physician density and PQIs was also unexpected. States with higher physician-per-capita rates had higher PQI scores in several categories, suggesting that a greater number of physicians does not necessarily correlate with better outcomes. This could reflect an imbalance between the availability of PCPs and specialists or insufficient care coordination. Future research should examine the distribution of physician types across states to better understand how the healthcare workforce composition affects population health.

Limitations

This study has several limitations. First, our analysis is cross-sectional, so we cannot infer causality. Second, we analyzed statewide data, which limits the representation of regional variability in healthcare access and outcomes. As noted above, geographic factors such as urbanization likely impact a patient’s access to care. Utilizing statewide data does not capture the healthcare infrastructure differences between, for example, urban counties versus rural counties within a state. Instead, it only reflects variation across states with more urbanization versus states with less urbanization. Additionally, our use of multivariable linear regression assumes a linear relationship between TCM utilization and PQI outcomes. This may overlook more complex dynamics, such as diminishing returns from TCM services at higher levels of implementation or nonlinear thresholds where the impact on hospitalizations plateaus. Furthermore, although we assessed multicollinearity and ensured model stability, the influence of outliers at the state level may have skewed the regression estimates. Future studies using patient-level or nonlinear modeling approaches (e.g., spline regression or mixed-effects models) may help address these nuances. Finally, our data are from 2022, which was a transitional period as individuals, communities, and healthcare systems were recovering from the effects of the COVID-19 pandemic. The pandemic interrupted access to care, so the quality outcomes and expected healthcare utilization rates may have been affected [44]. Therefore, the interpretation of this study should take into account the above limitations.

Future implications

Overall, our findings suggest that while strategic measures to improve continuity of care, such as TCM services, are promising for long-term patient outcomes, their success depends on integration into a broader framework of longitudinal care. In states with high PQIs, expanding access to TCM services may be necessary but insufficient. TCM services can serve as a meaningful touchpoint, but only when they are part of a sustained care relationship across settings, clinician interactions, and time. States with lower PQIs may be able to adopt TCM services not as a reactive measure, but as a proactive tool to reinforce care continuity systems. Policymakers and healthcare organizations should prioritize strategies that embed TCM services within a continuum of primary, specialty, and community-based care to ensure that transitions are not isolated events but part of an ongoing, coordinated effort to manage health over time.

Future studies building on this work could link patient-level hospital and outpatient data over time to evaluate whether continuity of care efforts, such as TCM services, are effectively reaching high-risk patients before avoidable hospitalizations occur. Additionally, it would be valuable to examine how other types of longitudinal care strategies, such as chronic care management participation, continuity with the same primary care team, and long-term coordination across specialties, impact patient outcomes and quality. Systemic barriers to continuity of care, such as long appointment wait times and limited transportation access, should also be analyzed. TCM is only one form of intervention to improve continuity of care, and it is important to capture additional quality indicators beyond inpatient discharge follow-up.

## Conclusions

Healthcare continuity is critical for effective, timely, and high-quality patient care. Gaps in continuous care can lead to adverse health outcomes, increased hospitalizations, and diminished patient trust in healthcare systems. This study aimed to assess whether states with increased follow-up after discharge also had lower rates of avoidable hospitalizations. We used multivariate linear regression to analyze the TCM-discharge ratio as a measure of follow-up care and PQIs as measures of hospitalizations that could have been avoided through outpatient care. We hypothesized that as TCM-discharge ratios increased, PQIs would decrease because states that utilized more continuity of care would have lower avoidable hospitalizations. Surprisingly, our findings showed the opposite: states with higher follow-up rates after discharge had higher PQI scores. This suggests that TCM services may be used more reactively to address hospitalizations rather than proactively to prevent them. Future research could build on this work by examining patient-level data across regions and over time to explore whether similar patterns hold for continuity of care services more broadly.

## Appendices

**Table 6:** Regression coefficients for Medicaid expansion status across prevention quality indicators (PQIs) *Indicates that physician density is statistically significant with PQIs at p < 0.05. Abbreviations: PQI, prevention quality indicator; COPD, chronic obstructive pulmonary disease; HRSA, Health Resources and Services Administration; TCM, transitional care management. Source: TCM and discharge data obtained from Centers for Medicare and Medicaid Services (CMS) [29]; PQI data obtained from CMS [30]; physician density rate obtained from HRSA [32].

Dependent Variable	B (unstandardized)	Standard Error	β (standardized)	t	Significance	Tolerance	Variance Inflation Factor (VIF)
PQI #1: Diabetes Short-Term Complications	-5.066	3.759	-0.149	-1.348	0.185	0.827	1.209
PQI #3: Diabetes Long-Term Complications	-9.364	12.929	-0.089	-0.724	0.473	0.827	1.209
PQI #5: COPD or Asthma in Older Adults	-4.216	30.467	-0.020	-0.138	0.891	0.827	1.209
PQI #7: Hypertension	-22.373	10.361	-0.232	-2.159	0.036*	0.827	1.209
PQI #8: Heart Failure	-81.001	67.527	-0.158	-1.200	0.237	0.827	1.209
PQI #11: Community-Acquired Pneumonia	-20.882	31.921	-0.071	-0.654	0.516	0.827	1.209
PQI #12: Urinary Tract Infection	-27.724	27.793	-0.110	-0.998	0.324	0.827	1.209
PQI #14: Uncontrolled Diabetes	-10.720	5.889	-0.213	-1.820	0.076	0.827	1.209
PQI #16: Lower-Extremity Amputation Among Patients with Diabetes	-4.795	5.094	-0.110	-0.941	0.352	0.827	1.209
PQI #90: Prevention Quality Overall Composite	-194.675	158.353	-0.147	-1.229	0.226	0.827	1.209
PQI #91: Prevention Quality Acute Composite	-48.452	55.218	-0.094	-0.887	0.385	0.827	1.209
PQI #92: Prevention Quality Chronic Composite	-144.125	112.864	-0.155	-1.277	0.208	0.827	1.209

**Table 7:** Regression coefficients for median age status across prevention quality indicators (PQIs) *Indicates that physician density is statistically significant with PQIs at p < 0.05. Abbreviations: PQI, prevention quality indicator; COPD, chronic obstructive pulmonary disease; CPT, Current Procedural Terminology; HRSA, Health Resources and Services Administration; TCM, transitional care management. Source: TCM and discharge data obtained from Centers for Medicare and Medicaid Services (CMS) [29]; PQI data obtained from CMS [30]; physician density rate obtained from HRSA [32].

Dependent Variable	B (unstandardized)	Standard Error	β (standardized)	t	Significance	Tolerance	Variance Inflation Factor (VIF)
PQI #1: Diabetes Short-Term Complications	-0.806	0.767	-0.123	-1.051	0.299	0.742	1.348
PQI #3: Diabetes Long-Term Complications	2.919	2.639	0.144	1.106	0.275	0.742	1.348
PQI #5: COPD or Asthma in Older Adults	3.173	6.218	0.076	0.510	0.612	0.742	1.348
PQI #7: Hypertension	-0.893	2.115	-0.048	-0.422	0.675	0.742	1.348
PQI #8: Heart Failure	11.703	13.782	0.118	0.849	0.401	0.742	1.348
PQI #11: Community-Acquired Pneumonia	-5.416	6.515	-0.096	-0.831	0.410	0.742	1.348
PQI #12: Urinary Tract Infection	0.032	5.672	0.001	0.006	0.996	0.742	1.348
PQI #14: Uncontrolled Diabetes	0.766	1.202	0.079	0.638	0.527	0.742	1.348
PQI #16: Lower-Extremity Amputation Among Patients with Diabetes	-3.163	1.040	-0.375	-3.042	0.004*	0.742	1.348
PQI #90: Prevention Quality Overall Composite	17.931	32.319	0.070	0.555	0.582	0.742	1.348
PQI #91: Prevention Quality Acute Composite	-5.437	11.270	-0.055	-0.482	0.632	0.742	1.348
PQI #92: Prevention Quality Chronic Composite	21.314	23.035	0.119	0.925	0.360	0.742	1.348

**Table 8:** Regression coefficients for HPSA need met across prevention quality indicators (PQIs) *Indicates that physician density is statistically significant with PQIs at p < 0.05. Abbreviations: PQI, prevention quality indicator; COPD, chronic obstructive pulmonary disease; HPSA, health professional shortage area; HRSA, Health Resources and Services Administration; TCM, transitional care management. Source: TCM and discharge data obtained from Centers for Medicare and Medicaid Services (CMS) [29]; PQI data obtained from CMS [30]; physician density rate obtained from HRSA [32].

Dependent Variable	B (unstandardized)	Standard Error	β (standardized)	t	Significance	Tolerance	Variance Inflation Factor (VIF)
PQI #1: Diabetes Short-Term Complications	0.106	0.114	0.099	0.934	0.355	0.907	1.103
PQI #3: Diabetes Long-Term Complications	-0.094	0.390	-0.028	-0.240	0.811	0.907	1.103
PQI #5: COPD or Asthma in Older Adults	-0.284	0.920	-0.042	-0.308	0.759	0.907	1.103
PQI #7: Hypertension	-0.134	0.313	-0.044	-0.429	0.670	0.907	1.103
PQI #8: Heart Failure	2.552	2.040	0.158	1.251	0.218	0.907	1.103
PQI #11: Community-Acquired Pneumonia	-0.427	0.964	-0.046	-0.443	0.660	0.907	1.103
PQI #12: Urinary Tract Infection	0.386	0.839	0.048	0.460	0.648	0.907	1.103
PQI #14: Uncontrolled Diabetes	0.067	0.178	0.042	0.379	0.707	0.907	1.103
PQI #16: Lower-Extremity Amputation Among Patients with Diabetes	-0.003	0.154	-0.002	-0.017	0.986	0.907	1.103
PQI #90: Prevention Quality Overall Composite	2.139	4.783	0.051	0.447	0.657	0.907	1.103
PQI #91: Prevention Quality Acute Composite	-0.064	1.668	-0.004	-0.038	0.970	0.907	1.103
PQI #92: Prevention Quality Chronic Composite	2.254	3.409	0.077	0.661	0.512	0.907	1.103

## References

[1] Fennelly O, Moroney D, Doyle M, Eustace-Cook J, Hughes M (2024). Key interoperability factors for patient portals and electronic health records: a scoping review. Int J Med Inform.

[2] Elysee G, Yu H, Herrin J, Horwitz LI (2021). Association between 30-day readmission rates and health information technology capabilities in US hospitals. Medicine (Baltimore).

[3] Gill JM, Mainous AG 3rd, Nsereko M (2000). The effect of continuity of care on emergency department use. Arch Fam Med.

[4] Surbhi S, Chen M, Shuvo SA (2022). Effect of continuity of care on emergency department and hospital visits for obesity-associated chronic conditions: a federated cohort meta-analysis. J Natl Med Assoc.

[5] Christakis DA, Mell L, Koepsell TD, Zimmerman FJ, Connell FA (2001). Association of lower continuity of care with greater risk of emergency department use and hospitalization in children. Pediatrics.

[6] Kraft KB, Hoff EH, Nylenna M, Moe CF, Mykletun A, Østby K (2024). Time is money: general practitioners' reflections on the fee-for-service system. BMC Health Serv Res.

[7] Timmins L, Kern LM, O'Malley AS, Urato C, Ghosh A, Rich E (2022). Communication gaps persist between primary care and specialist physicians. Ann Fam Med.

[8] Maughan BC, Lei L, Cydulka RK (2011). ED handoffs: observed practices and communication errors. Am J Emerg Med.

[9] Kripalani S, LeFevre F, Phillips CO, Williams MV, Basaviah P, Baker DW (2007). Deficits in communication and information transfer between hospital-based and primary care physicians: implications for patient safety and continuity of care. JAMA.

[10] Konrad R, Zhang W, Bjarndóttir M, Proaño R (2019). Key considerations when using health insurance claims data in advanced data analyses: an experience report. Health Syst (Basingstoke).

[11] Ferver K, Burton B, Jesilow P (2009). The use of claims data in healthcare research. Open Public Health J.

[12] Hsiang WR, Gross CP, Maroongroge S, Forman HP (2020). Trends in compensation for primary care and specialist physicians after implementation of the Affordable Care Act. JAMA Netw Open.

[13] Reid RO, Tom AK, Ross RM, Duffy EL, Damberg CL (2022). Physician compensation arrangements and financial performance incentives in US health systems. JAMA Health Forum.

[14] Pacheco J, Cuadrado C, Martínez-Gutiérrez MS (2019). Urgent care centres reduce emergency department and primary care same-day visits: a natural experiment. Health Policy Plan.

[15] Wang MC, Ryan G, McGlynn EA, Mehrotra A (2010). Why do patients seek care at retail clinics, and what alternatives did they consider?. Am J Med Qual.

[16] Catapan SC, Sazon H, Zheng S, Gallegos-Rejas V, Mendis R, Santiago PH, Kelly JT (2025). A systematic review of consumers' and healthcare professionals' trust in digital healthcare. NPJ Digit Med.

[17] Levine DM, Linder JA (2016). Retail clinics shine a harsh light on the failure of primary care access. J Gen Intern Med.

[18] Scott DR, Batal HA, Majeres S, Adams JC, Dale R, Mehler PS (2009). Access and care issues in urban urgent care clinic patients. BMC Health Serv Res.

[19] (2025). Alternative sites for health care. https://www.healthyagingpoll.org/reports-more/report/alternative-sites-health-care.

[20] Ljungholm L, Klinga C, Edin-Liljegren A, Ekstedt M (2022). What matters in care continuity on the chronic care trajectory for patients and family carers?—A conceptual model. J Clin Nurs.

[21] Pandhi N, Saultz JW (2006). Patients' perceptions of interpersonal continuity of care. J Am Board Fam Med.

[22] Jones KC, Austad K, Silver S (2023). Patient perspectives of the hospital discharge process: a qualitative study. J Patient Exp.

[23] Gurewich D, Garg A, Kressin NR (2020). Addressing social determinants of health within healthcare delivery systems: a framework to ground and inform health outcomes. J Gen Intern Med.

[24] Johnston KJ, Mittler J, Hockenberry JM (2020). Patient social risk factors and continuity of care for medicare beneficiaries. Health Serv Res.

[25] Mixon AS, Goggins K, Nwosu S (2024). Association of social determinants of health with hospital readmission and mortality: a prospective cohort study. Health Lit Res Pract.

[26] Feldman SS, Davlyatov G, Hall AG (2020). Toward understanding the value of missing social determinants of health data in care transition planning. Appl Clin Inform.

[27] Joo JY (2023). Fragmented care and chronic illness patient outcomes: a systematic review. Nurs Open.

[28] Rangachari P, Thapa A (2025). Impact of hospital and health system initiatives to address social determinants of health (SDOH) in the United States: a scoping review of the peer-reviewed literature. BMC Health Serv Res.

[29] (2025). Medicare physician & other practitioners - by provider and service. https://data.cms.gov/provider-summary-by-type-of-service/medicare-physician-other-practitioners/medicare-physician-other-practitioners-by-provider-and-service.

[30] (2025). Mapping medicare disparities by population. https://data.cms.gov/tools/mapping-medicare-disparities-by-population.

[31] (2025). Status of state medicaid expansion decisions. https://www.kff.org/status-of-state-medicaid-expansion-decisions/.

[32] (2025). Area health resources files. https://data.hrsa.gov/topics/health-workforce/ahrf.

[33] U.S. Census Bureau (2025). Poverty status in the past 12 months. https://data.census.gov/table/ACSST1Y2023.S1701?q=Population+for+whom+poverty+status+is+determined&g=010XX00US$0400000.

[34] U.S. Census Bureau (2025). Urban and rural. https://data.census.gov/table/DECENNIALCD1182020.P2?q=urban+and+rural+116th.

[35] U.S. Census Bureau (2025). Age and sex. https://data.census.gov/table/ACSST1Y2022.S0101?q=median+age&g=010XX00US$0400000&y=2022&moe=false.

[36] (2025). Health workforce shortage areas. https://data.hrsa.gov/topics/health-workforce/shortage-areas.

[37] (2025). Transitional care management services. https://www.cms.gov/files/document/mln908628-transitional-care-management-services.pdf.

[38] Bindman AB, Cox DF (2018). Changes in health care costs and mortality associated with transitional care management services after a discharge among medicare beneficiaries. JAMA Intern Med.

[39] Tyler N, Hodkinson A, Planner C (2023). Transitional care interventions from hospital to community to reduce health care use and improve patient outcomes: a systematic review and network meta-analysis. JAMA Netw Open.

[40] Kim EJ, Coppa K, Abrahams S, Hanchate AD, Mohan S, Lesser M, Hirsch JS (2025). Utilization of transitional care management services and 30-day readmission. PLoS One.

[41] (2025). The ins and outs of transitional care management coding. https://www.mgma.com/articles/the-ins-and-outs-of-transitional-care-management-coding.

[42] (2025). Prevention quality indicators in inpatient settings measures. https://qualityindicators.ahrq.gov/measures/pqi_resources.

[43] Zhang Z, Zhao M, Zhang Y, Feng Y (2023). How does urbanization affect public health? New evidence from 175 countries worldwide. Front Public Health.

[44] Matenge S, Sturgiss E, Desborough J, Hall Dykgraaf S, Dut G, Kidd M (2022). Ensuring the continuation of routine primary care during the COVID-19 pandemic: a review of the international literature. Fam Pract.

